# Homocysteine, cysteine and methionine are associated with 90-day prognosis in intracerebral hemorrhage patients

**DOI:** 10.1080/07853890.2026.2719236

**Published:** 2026-08-21

**Authors:** Ming Fang, Tianliang Xia, Yan Yin

**Affiliations:** Department of Neurology, The Second Hospital of Dalian Medical University, Dalian, China

**Keywords:** Homocysteine, cysteine, methionine, intracerebral hemorrhage, prognosis

## Abstract

**Objectives:**

This retrospective study explored the associations of plasma homocysteine (Hcy), cysteine (Cys) and methionine (Met) levels with 90-day prognosis in intracerebral hemorrhage (ICH) patients and evaluated their predictive value for ICH prognosis.

**Methods:**

A total of 212 ICH patients and 215 age- and sex-matched healthy controls were enrolled. ICH patients were categorized into favorable and unfavorable prognostic groups based on their 90-day prognosis. Plasma Hcy, Cys and Met levels were compared between groups. Multivariate logistic regression and ROC curve analyses were used to assess their prognostic correlations and predictive efficacy. Restricted Cubic Spline (RCS) analysis explored the nonlinear relationship between Met and prognosis. The predictive model was internally validated and compared with classic ICH prognostic scoring systems.

**Results:**

ICH patients had significantly higher Hcy, Cys and Met levels than healthy controls. Unfavorable prognosis patients presented higher Hcy and Cys levels but lower Met levels. High Hcy (OR = 1.235, 95%CI 1.137–1.342) and Cys (OR = 1.017, 95%CI 1.008–1.025) were independent risk factors for poor prognosis. Met exerted a dual nonlinear effect and played a protective role at levels <32.88 μmol/L (OR = 0.924, 95%CI 0.860–0.992). The combined three biomarkers yielded the optimal predictive performance (AUC = 0.819), superior to all single and pairwise biomarker combinations.

**Conclusion:**

Elevated Hcy, Cys and Met levels are observed in ICH patients. High Hcy and Cys predict unfavorable prognosis. Met exhibited concentration-dependent correlational trends, lower odds of adverse outcomes were observed for Met <32.88 μmol/L. Combined detection of the three biomarkers yielded the highest predictive accuracy in internal single-center validation, pending external prospective verification before clinical translation.

## KEY MESSAGES

- Plasma levels of Hcy, Cys and Met are associated with 90-day prognosis after intracerebral hemorrhage.
- Triple combination of Hcy, Cys and Met has greater predictive value, providing a more comprehensive risk assessment of unfavorable prognosis in ICH patients.

## Introduction

Intracerebral hemorrhage (ICH) is a critical subtype of stroke, marked by its abrupt onset, rapid progression, and high rates of mortality and disability [1,2]. Although ICH accounts for only 10–20% of all stroke cases [3], it contributes to 44.1% of stroke-related mortality [4]. Only 20% of ICH patients achieve functional independence [5]. Age, gender and race are nonmodifiable risk factors for ICH. Factors that can be modified include hypertension, diabetes mellitus, smoking, alcohol consumption and use of anticoagulant or antiplatelet medications [6–8].

At present, commonly used prognostic evaluation tools for ICH mainly rely on clinical scoring systems such as ICH score, NIHSS and GCS, which are limited by subjective assessment and insufficient sensitivity for early risk stratification. Peripheral blood biomarkers are expected to realize objective, rapid and early prognostic judgment, but high-efficiency combined biomarker panels are still lacking in clinical practice. Sulfur amino acids including Hcy, Cys and Met participate in cerebrovascular injury, oxidative stress and neuroinflammation, which are core pathological mechanisms of ICH. Considering their close metabolic linkage and synergistic biological effects, we hypothesized that combined detection of the three indexes could make up for the deficiency of single biomarker and traditional clinical scores, and improve the accuracy of prognostic prediction for ICH. This study aimed to fill the research gap of combined application of the three sulfur amino acids in ICH prognostic evaluation.

**Figure 1.**
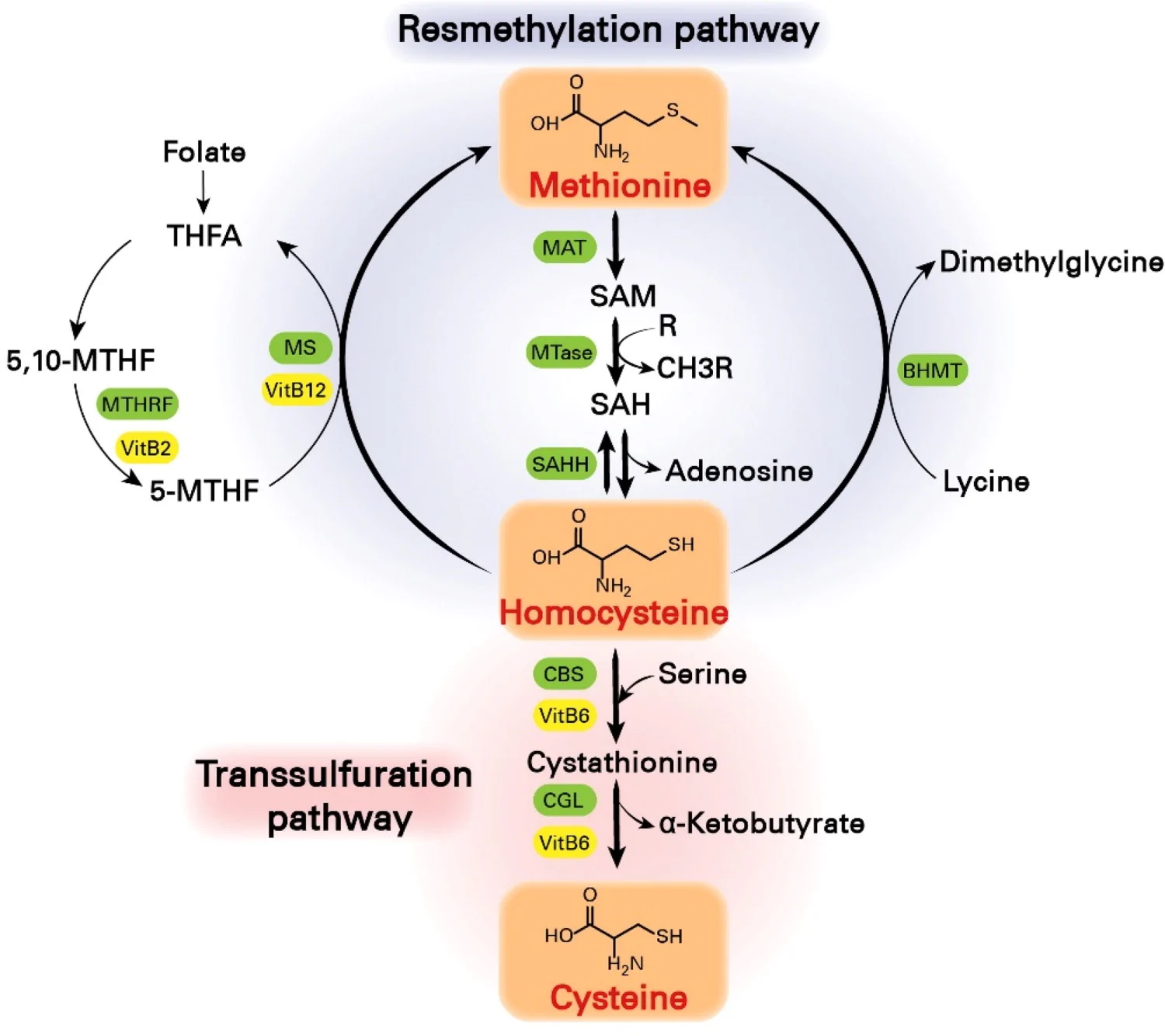
Metabolic pathways of homocysteine, cysteine and methionine. This figure displays two core metabolic pathways of three sulfur amino acids: Remethylation pathway (Met transforms to Hcy, and Hcy is reconverted to Met with the participation of folate, vitamin B12 and related enzymes) and Transsulfuration pathway (Hcy generates Cys under the action of vitamin B6 and key enzymes). THFA: tetrahydrofolic acid; 5,10-MTHF: 5, 10-methylenetetrahydrofolate; MS: methionine synthase; MTHFR: 5,10-methylenetetrahydrofolate reductase; 5-MTHF: 5-methyltetrahydrofolate; MAT: methionine adenosyltransferase; SAM: S-adenosylmethionine; MTase: methyltransferase; SAH: S-adenosine homocysteine; SAHH: homocysteine hydrolase; BHMT: Betaine-homocysteine methyl-transferase; CBS: cystathionine β-synthase; CGL: cystathionine γ-lyase.

Increased concentrations of homocysteine (Hcy) are linked to an increased risk of various neurological disorders, including ICH [9], ischemic stroke [10], Moyamoya Disease [11], Alzheimer’s disease [12], multiple sclerosis [13] and Parkinson’s disease [14]. Hyperhomocysteinemia is a known risk factor for ICH and relates to its severity and poor prognosis [15,16]. Cysteine (Cys) is critically involved in maintaining protein structural integrity and antioxidative defense [17]. Cysteine-altering NOTCH3 variants are a risk factor for cerebral small vessel disease [18]. Methionine (Met) is an essential amino acid and serves as a precursor in the Hcy biosynthetic pathway [19]. It may play a role in the prevention of cerebrovascular diseases [20], but its effect on ICH remains unconfirmed. The sulfur amino acids (SAA) Hcy, Cys and Met are interconverted, as shown in Figure 1. Hcy is produced from Met *via* S-adenosylmethionine (SAM) and S-adenosylhomocysteine (SAH). It can then be remethylated back to Met through vitamin B12-dependent methionine synthase or betaine-homocysteine methyltransferase. Alternatively, Hcy enters the transsulfuration pathway, where it is condensed with serine by vitamin B6-dependent cystathionine β-synthase to form cystathionine, which is then cleaved by cystathionine γ-lyase into Cys and α-ketobutyrate [21]. However, the collective impact of these three SAAs on ICH prognosis has not been elucidated. Therefore, our study aimed to systematically investigate the associations of Hcy, Cys, Met levels with clinical prognosis in ICH patients, and to evaluate their predictive value. Our research may provide preliminary exploratory insights for predicting the prognosis of ICH.

## Methods

### Study design

Sample size calculation was performed retrospectively after patient enrollment based on the expected OR value of main exposure indicators, α = 0.05, test power (1-β) =0.80. Calculation was conducted using standard logistic regression sample size formula in PASS 15.0 software, with assumptions of an unfavorable outcome event rate of 42% among ICH patients and an expected OR of 1.3 for plasma Hcy. The calculated minimum sample size for ICH patients was 190 cases, and we finally enrolled 212 ICH patients, which fully met the requirement of multivariate statistical analysis and avoided statistical bias caused by insufficient sample size. As this evaluation was conducted post hoc, it requires cautious interpretation pending external validation.

This study included a cohort of 212 consecutive patients diagnosed with primary intracerebral hemorrhage (ICH), all of whom were admitted to the Neurology Department at the Second Hospital of Dalian Medical University during the period from September 2022 to September 2024. The ICH group included individuals with an initial diagnosis of acute ICH [22] who completed Hcy, Cys and Met assessments within 24 h of hospitalization. Exclusion criteria were as follows: (1) Secondary ICH caused by trauma, brain tumor, aneurysm, vascular malformation, hematologic disease, or use of oral anticoagulants; (2) Use of folate, vitamin B6, or vitamin B12 supplements within 3 months; (3) Patients receiving surgical intervention (intracranial hematoma evacuation, lateral ventricular drainage, or cerebrospinal fluid diversion) were excluded. This group represents critically severe ICH patients with extremely high mortality, and excluding them can reduce the interference of extreme cases on biomarker analysis; (4) Concurrent severe cardiovascular, hepatic, renal diseases, or malignant tumors; (5) Lost to follow-up. 215 age- and sex-matched healthy individuals recruited during the same period were included in control group. Control subjects were excluded if they had a history of stroke or met any of the exclusion criteria applied to the ICH group.

This study adopted a two-part research design: the case-control part compared the differences of three sulfur amino acids between ICH patients and healthy people to explore their correlation with ICH onset; the prognostic analysis part focused on ICH patients alone to analyze the relationship between biomarkers and 90-day functional prognosis. The two research objectives were independent and complementary, and the rationality of the design was guaranteed.

The Institutional Ethics Review Board at the Second Hospital of Dalian Medical University granted approval for this research protocol (Ethics Approval Code: KY2024-455-01). Given the retrospective design of this study and the anonymization of data, written informed consent was not obtained during the study procedure, and all patient data were stripped of personal identifiers prior to analysis. The study procedures adhered to the ethical principles outlined in the Helsinki Declaration.

### Data collection

Patient clinical information was recorded, including the following: (1) baseline demographics (gender, age); (2) comorbidity profiles (hypertension, diabetes mellitus); (3) behavioral factors (smoking, alcohol consumption); (4) neuroimaging characteristics (hematoma location and volumetric measurements). For biochemical analysis, fasting venous blood samples (2–3 mL) were obtained. Considering that acute stress after cerebral hemorrhage may affect the level of peripheral blood amino acids, we uniformly collected blood samples in the resting state of patients within 24 h after admission, excluding patients with acute infection, shock and other severe stress states. The levels of Hcy, Cys and Met were quantified using liquid chromatography-mass spectrometry (LC-MS).

### Follow-up and prognosis

Two board-certified neurologists, blinded to baseline clinical and prognostic data, conducted standardized follow-ups 90 days after discharge *via* telephone interviews or outpatient visits. The participants were stratified into two prognostic groups according to their mRS score: the favorable prognosis group (mRS ≤ 2) and the unfavorable prognosis group (mRS > 2).

### Statistical analysis

Statistical analyses and data plotting were performed using IBM SPSS (Version 26.0), GraphPad Prism (Version 10.0) and R (Version 4.5.2). Categorical variables were summarized as frequencies and percentages, with intergroup comparisons made using chi-square tests. The normality of continuous data was assessed with the Shapiro-Wilk test separately within the overall ICH-control pooled cohort and within each stratified prognostic subgroup. Data conforming to a normal distribution were compared using independent t-tests and expressed as mean ± standard deviation (SD), whereas non-normally distributed data were compared using the Mann-Whitney U test and reported as median and interquartile range (IQR). Spearman’s correlation coefficient was used to examine the correlations between the variables and ICH prognosis.

Predictors associated with ICH prognosis were identified through multivariable logistic regression. Model building strategy: firstly, all variables with *p* < 0.05 in univariate analysis were included; then stepwise regression was used to screen independent influencing factors. Covariates fully adjusted in the model included age, gender, hypertension, diabetes, smoking, alcohol consumption, hematoma location, hematoma volume. Variance Inflation Factor (VIF) was used to test multicollinearity, and VIF < 2 was defined as no obvious multicollinearity. We strictly controlled the ratio of events to variables to avoid model overfitting. Results of regression were presented as odds ratios (ORs) and their 95% confidence intervals (CIs). Restricted cubic spline (RCS) analysis was applied to explore potential non-linear relationships.

Furthermore, the predictive performance of Hcy, Cys and Met was evaluated by receiver operating characteristic (ROC) curve analysis, quantified by the area under the curve (AUC). Bootstrap internal validation (1000 resampling) was performed for all ROC models, and calibration curve was drawn to evaluate model calibration (Hosmer-Lemeshow test). Meanwhile, we compared the predictive efficiency of the triple-biomarker model with the classic ICH prognostic score using DeLong test. Decision curve analysis (DCA) was used to assess the net clinical benefit of the combined biomarker model and the classic ICH prognostic score. The optimal cutoff values, determined by maximizing the Youden index, were used to define the greatest prognostic prediction accuracy.

Biomarker quartile grouping according to the overall distribution of Hcy, Cys and Met in 212 ICH patients, interquartile segmentation was adopted to divide all cases into four groups. The quartile cutoffs were determined based on the actual measured values of the study population, which is a common standardized grouping method in clinical statistics, and the specific cut-off points were explained in detail in the text.

The statistical significance level was *p* < 0.05.

## Results

### Baseline characteristics

**Figure 2.**
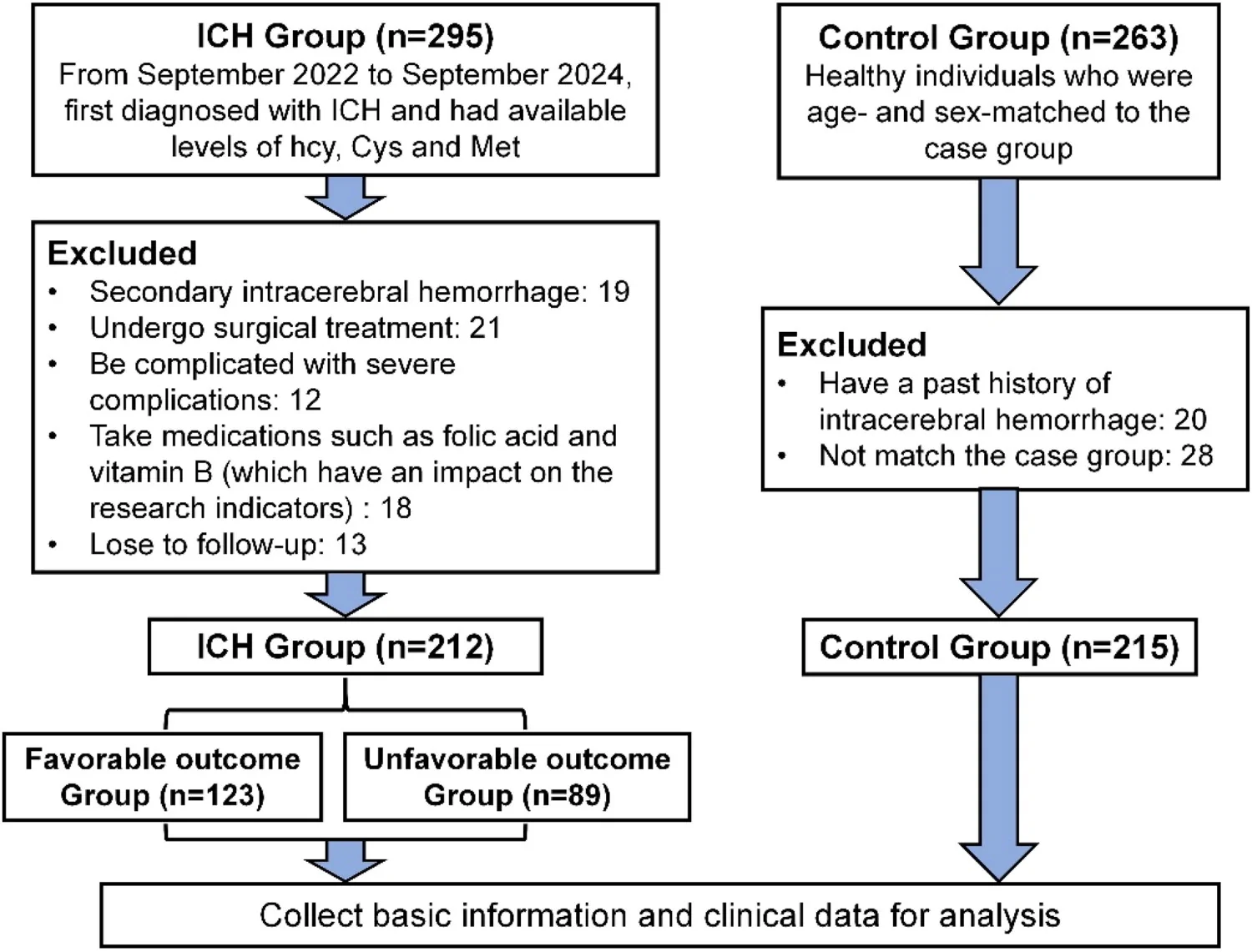
Flow diagram showing the enrollment of ICH patients and healthy controls. ICH: intracerebral hemorrhage.

Our study ultimately included a total of 212 acute intracerebral hemorrhage patients, with 123 cases in the 90-day favorable prognosis group and 89 cases in the poor prognosis group. The concurrent control group comprised 215 age- and sex-matched healthy individuals, as shown in Figure 2.

Comparative analysis indicated a significant disparity in hypertension rates between ICH and control groups (*p* < 0.05) and comparable distributions of gender, age, diabetes history, smoking and alcohol consumption (*p* > 0.05). Compared with the controls, the ICH group presented significantly elevated levels of Hcy, Cys and Met (*p* < 0.05), as shown in Table 1.

**Table 1.** Baseline clinical traits of ICH patients and healthy controls.

Variables	ICH (*n* = 212)	Control (*n* = 215)	*p* Value
Age (years)	62.76 ± 14.21	62.68 ± 13.10	0.949
Gender, *n* (%)			0.708
Male	129 (60.8)	127 (59.1)	
Female	83 (39.2)	88 (40.9)	
Medical History			
Hypertension, *n* (%)			<0.001
Yes	172 (81.1)	89 (41.4)	
No	40 (18.9)	126 (58.6)	
Diabetes, *n* (%)			0.663
Yes	44 (20.8)	41 (19.1)	
No	168 (79.2)	174 (80.9)	
Personal History			
Smoking, *n* (%)			0.590
Yes	61 (28.8)	67 (31.2)	
No	151 (71.2)	148 (68.8)	
Drinking, *n* (%)			0.441
Yes	52 (24.5)	46 (21.4)	
No	160 (75.5)	169 (78.6)	
Laboratory indicators			
Hcy (μmol/L)	11.77 (9.13,16.03)	8.43 (7.21,9.93)	<0.001
Cys (μmol/L)	246.38 (219.33,278.53)	231.91 (212.87,248.80)	<0.001
Met (μmol/L)	20.92 (16.69,24.39)	19.01 (16.73,22.11)	0.016

Hcy, homocysteine; Cys, cysteine; Met, methionine.

**Figure 3.**
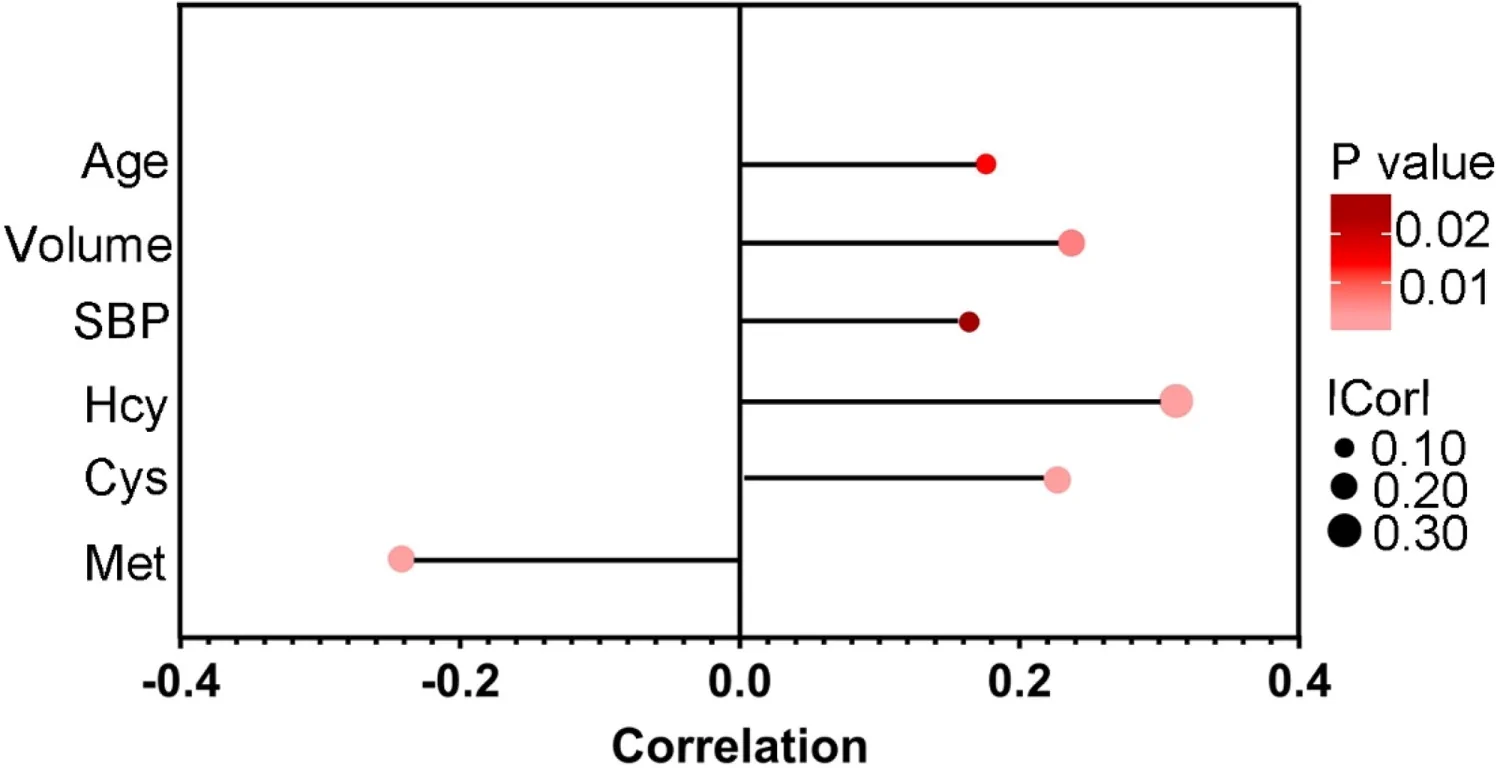
Correlation analysis graph of 90-day mRS scores after ICH (Spearman’s test). SBP: systolic blood pressure; Hcy: homocysteine; Cys: cysteine; Met: methionine.

Correlation analysis showed that age (*r* = 0.176, *p* = 0.01), hematoma volume (*r* = 0.237, *p* = 0.001), systolic blood pressure (*r* = 0.164, *p* = 0.017), Hcy (*r* = 0.312, *p* < 0.001) and Cys (*r* = 0.227, *p* < 0.001) were positively correlated with 90-day mRS scores after ICH, while Met (r = −0.242, *p* < 0.001) was negatively correlated (Figure 3).

### Comparative analysis of the prognosis of ICH patients

Patients with ICH were followed up for 90 days and classified into two groups based on the mRS score; 123 patients were in the favorable prognosis group and 89 in the unfavorable prognosis group.

Comparative analysis revealed that the mean age in the unfavorable prognosis group (65.62 ± 16.45 years) exceeded that of the favorable prognosis group (60.70 ± 11.99 years) (*p* < 0.05). A significant difference in hematoma volume was observed between the two groups (*p* < 0.05), with larger hemorrhage volumes mainly found in the unfavorable prognosis group. No notable differences were detected in the remaining baseline clinical variables across groups, including gender, hypertension history, diabetes history, smoking, drinking and hemorrhage location (*p* > 0.05), as shown in Table 2.

**Table 2.** Comparative analysis of baseline characteristics between the favorable and unfavorable prognosis groups in ICH patients.

Variables	Favorable prognosis (*n* = 123)	Unfavorable prognosis (*n* = 89)	*p* Value
Age, mean ± SD, year	60.7 ± 11.99	65.62 ± 16.45	0.018
Gender, *n* (%)			0.965
Male	75 (61.0)	54 (60.7)	
Female	48 (39.0)	35 (39.3)	
Medical History			
Hypertension, *n* (%)			0.524
Yes	98 (79.7)	74 (83.1)	
No	25 (20.3)	15 (16.9)	
Diabetes, *n* (%)			0.600
Yes	24 (19.5)	20 (22.5)	
No	99 (80.5)	69 (77.5)	
Personal History			
Smoking, *n* (%)			0.852
Yes	36 (29.3)	25 (28.1)	
No	87 (70.7)	64 (71.9)	
Drinking, *n* (%)			0.956
Yes	30 (24.4)	22 (24.7)	
No	93 (75.6)	67 (75.3)	
Neuroimaging Characteristics			
Location, *n* (%)			0.301
Supratentorial	106 (86.2)	72 (80.9)	
Infratentorial	17 (13.8)	17 (19.1)	
Volume, *n* (%)			<0.001
≥30 mL	13 (10.6)	32 (36.0)	
<30 mL	110 (89.4)	57 (64.0)	
Laboratory indicators			
Hcy, mean ± SD, μmol/L	10.85 ± 3.58	16.79 ± 7.58	<0.001
Hcy, *n* (%)			<0.001
Quartile1 (<9.13)	41 (33.3)	12 (13.5)	
Quartile2 (9.13–11.77)	41 (33.3)	12 (13.5)	
Quartile3 (11.77–16.03)	33 (26.7)	20 (22.5)	
Quartile4 (>16.03)	8 (6.5)	45 (50.5)	
Cys, mean ± SD, μmol/L	236.69 ± 42.62	275.99 ± 58.34	<0.001
Cys, *n* (%)			<0.001
Quartile1 (<219.33)	39 (31.7)	14 (15.7)	
Quartile2 (219.33–246.38)	39 (31.7)	14 (15.7)	
Quartile3 (246.38–278.53)	29 (23.6)	24 (27.0)	
Quartile4 (>278.53)	16 (13.0)	37 (41.6)	
Met, mean ± SD, μmol/L	22.00 ± 5.01	18.59 ± 5.98	<0.001
Met, *n* (%)			0.001
Quartile1 (<16.69)	20 (16.3)	33 (37.1)	
Quartile2 (16.69–20.92)	29 (23.6)	25 (28.1)	
Quartile3 (20.92–24.39)	34 (27.6)	18 (20.2)	
Quartile4 (>24.39)	40 (32.5)	13 (14.6)	

Hcy: homocysteine; Cys: cysteine; Met: methionine.

**Figure 4.**
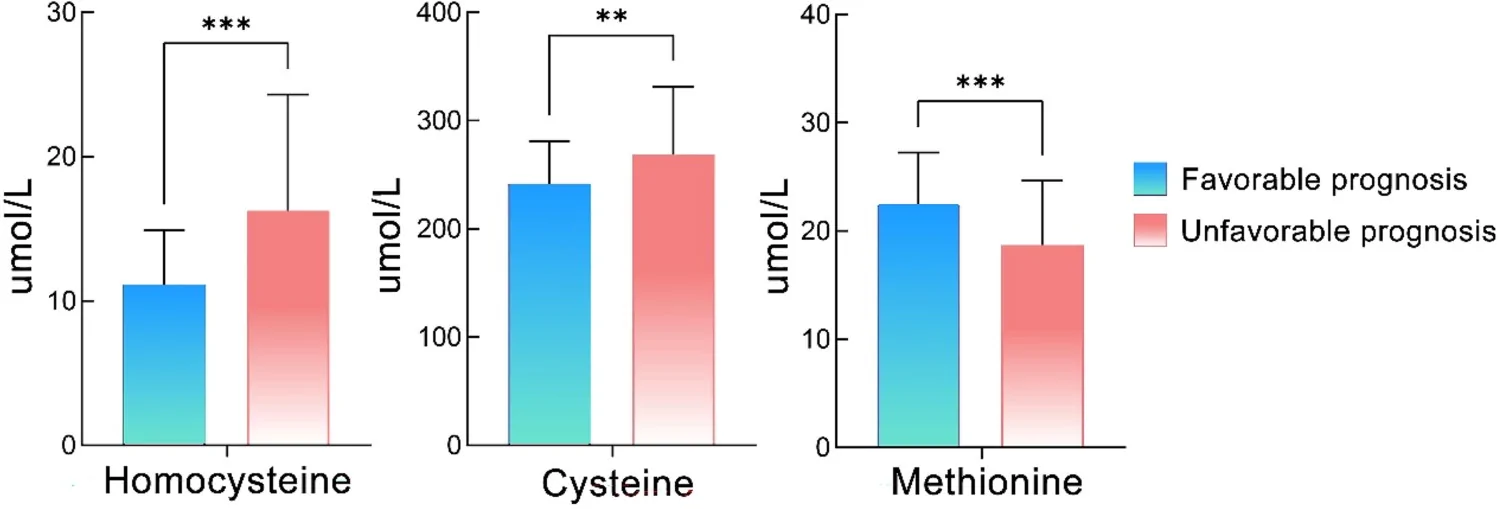
Comparison of homocysteine, cysteine and methionine levels between the favorable and unfavorable prognosis groups. *p < 0.05, **p < 0.01, ***p < 0.001.

Plasma levels of Hcy and Cys were substantially elevated in the unfavorable prognosis group (Hcy: 16.79 ± 7.58 vs. 10.85 ± 3.58 μmol/L, Cys: 275.99 ± 58.34 vs. 236.69 ± 42.62 μmol/L, *p* < 0.05). Conversely, the Met level demonstrated an inverse relationship, with a significant reduction in the unfavorable prognosis group (18.59 ± 5.98 vs. 22.00 ± 5.01 μmol/L, *p* < 0.05), as shown in Table 2 and Figure 4.

Hcy, Cys and Met levels were divided into quartiles (Q1–Q4) based on their distributions in the ICH individuals. Quartile grouping was conducted according to the interquartile range of all enrolled ICH patients, which is a standard grouping method for analyzing the graded risk of continuous variables. The quartile cutoffs were determined as follows: Hcy: Q1 ≤ 9.13 μmol/L, Q2 9.13–11.77 μmol/L, Q3 11.77–16.03 μmol/L and Q4 > 16.03 μmol/L; Cys: Q1 ≤ 219.33 μmol/L, Q2 219.33–246.38 μmol/L, Q3 246.38–278.53 μmol/L and Q4 > 278.53 μmol/L; Met: Q1 ≤ 16.69 μmol/L, Q2 16.69–20.92 μmol/L, Q3 20.92–24.39 μmol/L and Q4 > 24.39 μmol/L. The lowest quartile (Q1) for each biomarker was designated as the reference group to assess risk gradients associated with higher exposure levels. Quartile distribution analyses revealed significant intergroup differences among three amino acids (all *p* < 0.05). Patients with unfavorable prognoses disproportionately clustered in upper quartiles (quartile 4) of Hcy (50.5%) and Cys (41.6%), and in lower quartiles (Q 1) of Met (37.1%), as shown in Table 2.

### Univariate and multivariate logistic regression analysis of ICH prognosis

**Figure 5.**
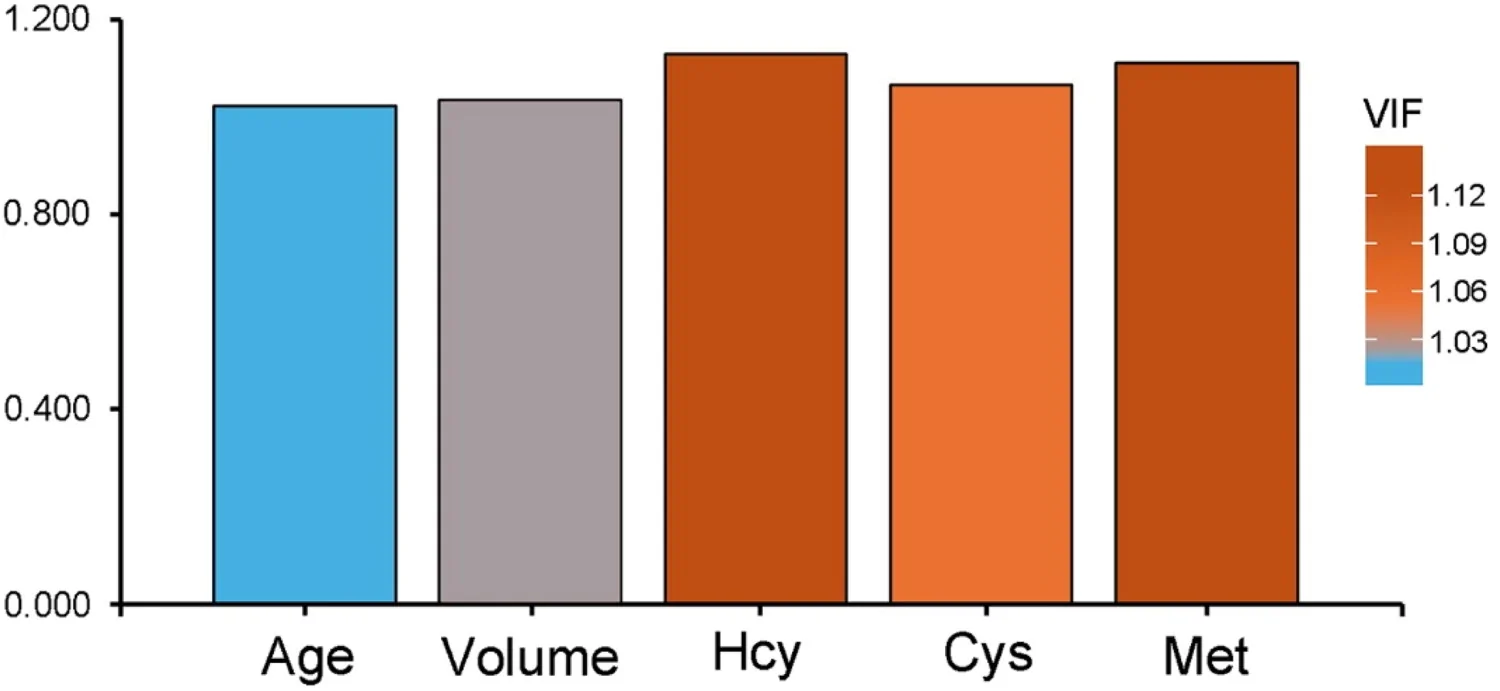
Variance inflation factors for variables significant in univariate analysis. The figure shows the specific VIF values of each variable: Hcy = 1.12, Cys = 1.09, Met = 1.06, Age = 1.03, Volume = 1.20. All VIF values < 2.0, suggesting no multicollinearity. Hcy: homocysteine; Cys: cysteine; Met: methionine.

To elucidate the relationship between variables significant in univariate analysis and the prognosis of ICH, univariate logistic regression analysis was performed. To adjust for potential confounding factors, multivariate logistic regression analysis was subsequently conducted. Prior to this multivariate analysis, variables demonstrating statistical significance in the univariate analysis were selected and assessed for collinearity. The Variance Inflation Factor (VIF) for all selected variables ranged between 1.0 and 1.2, which was far lower than 2.0, indicating no severe multicollinearity (Figure 5). Therefore, the regression coefficient estimates were stable and reliable, and the independent effects of these variables could be effectively interpreted.

**Figure 6.**
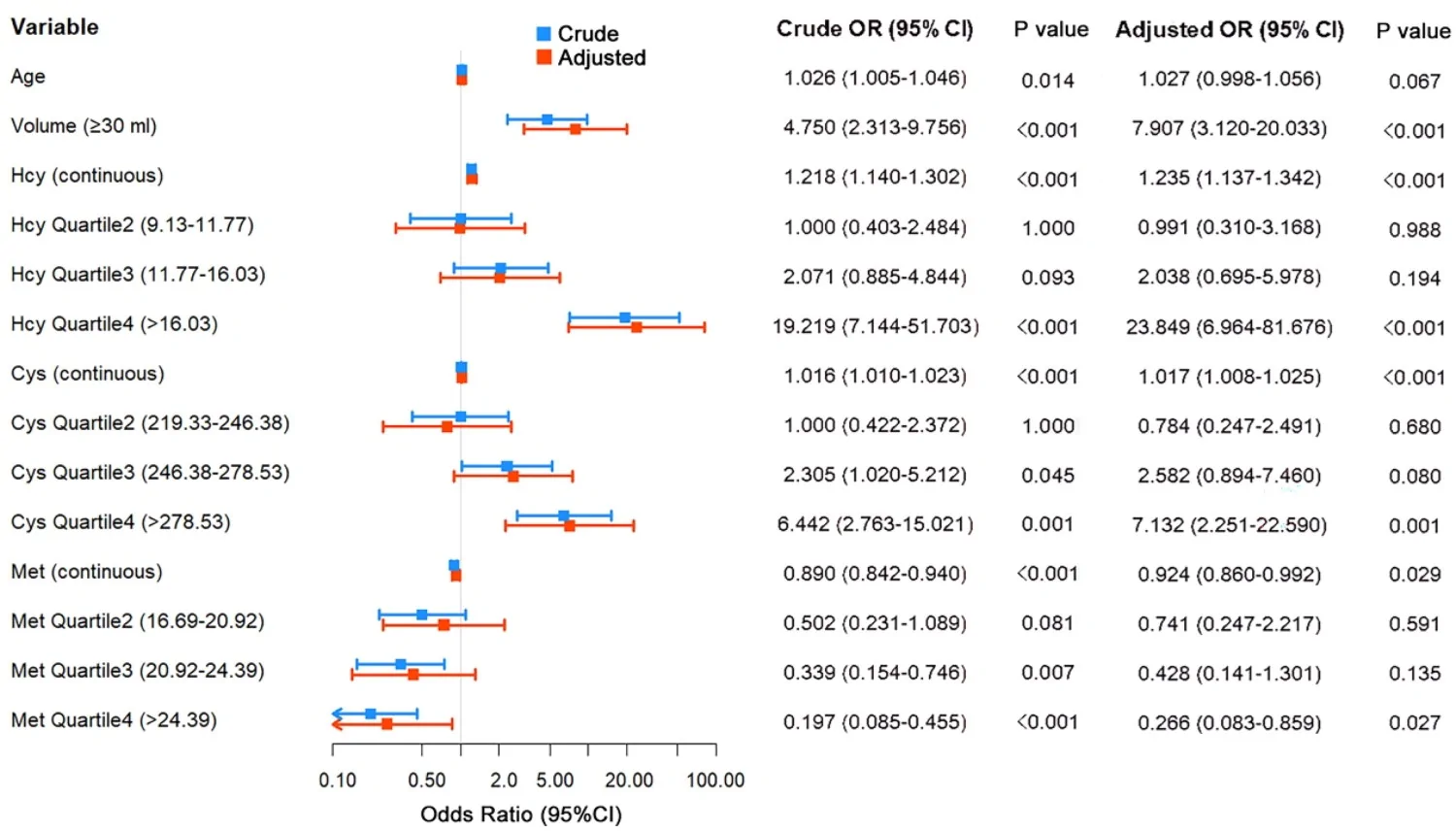
Logistic analysis and Forest Plot of predictors of prognosis in patients with ICH. Adjusted for age, gender, hypertension, diabetes, smoking, drinking, hemorrhage location and hemorrhage volume. Hcy: homocysteine; Cys: cysteine; Met: methionine.

As shown in Figure 6, unadjusted Hcy levels, treated as continuous variables, were correlated with a 21.8% increased risk of unfavorable prognosis per unit. This risk further increased to 23.5% after adjustment. Each 1 μmol/L increase in plasma Hcy was associated with 23.5% higher odds of an unfavorable 90-day prognosis. Among the categorical variables, only the highest quartile (Q4 > 16.03 μmol/L) was associated with a significant risk, with an adjusted OR of 23.849. The high OR value of Hcy in the highest quartile was related to the obvious concentration gradient of Hcy in patients with poor prognosis. We have fully adjusted multiple confounding factors in the model to reduce residual confounding; meanwhile, the sample distribution of this group was concentrated, which also led to relatively high OR value. This finding should be interpreted as an observational association rather than a causal effect. After adjusting for confounding variables, no significant associations were found in the other quartiles.

Analyses of continuous measures showed that non-adjusted Cys levels had a 1.6% higher likelihood of adverse clinical outcomes. Each 1 μmol/L increase in plasma Cys was associated with 1.7% higher odds of an unfavorable prognosis after adjustment. The increased risk was significant only in the highest quartile (Q4 > 278.53 μmol/L), with an adjusted OR of 7.132 (Figure 6).

In the unadjusted model, each unit increase in Met levels conferred an 11.0% reduction in the risk of an unfavorable prognosis. Each 1 μmol/L increase in plasma Met was associated with 7.6% lower odds of an unfavorable prognosis after adjustment. The most pronounced protective association was observed in the highest quartile (Q4 > 24.39 μmol/L), with an adjusted OR of 0.266 (Figure 6).

**Figure 7.**
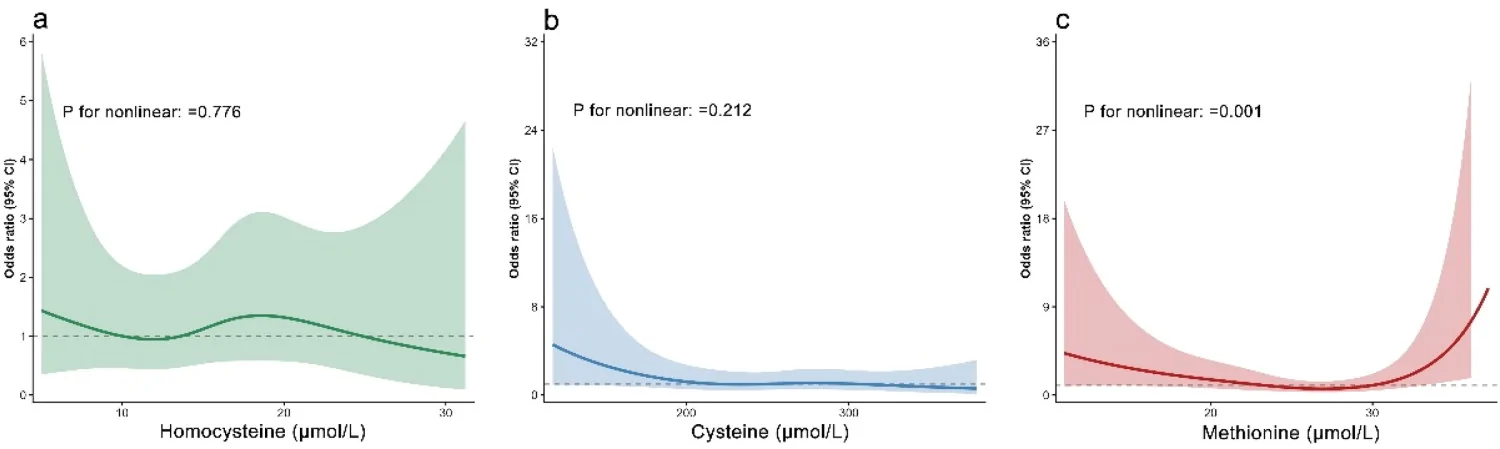
Restricted cubic spline analysis (adjusted model) for association of homocysteine (a), cysteine (b) and methionine (c) with ICH prognosis. Adjusted for age, gender, hypertension, diabetes, smoking, drinking, hemorrhage location and hemorrhage volume.

Upon applying Restricted Cubic Spline (RCS) regression adjusted for confounders, a nonlinear association was identified between Met level and ICH prognosis (nonlinear *p*-values < 0.05, Figure 7). The exploratory inflection point derived from this single-centre retrospective cohort was 32.88 μmol/L. Only 3 patients in the full ICH sample presented Met concentrations above this cutoff value. When dichotomized by 32.88 μmol/L, the unadjusted odds ratio for adverse outcomes among patients exceeding this threshold was 2.80 (95%CI 0.25–31.42, *p* = 0.403). A numerical upward trend in poor outcome odds was observed for Met concentrations above 32.88 μmol/L, yet the association lacked statistical significance. For Met concentrations below 32.88 μmol/L, higher Met levels were continuously correlated with reduced odds of unfavorable functional outcomes. This 32.88 μmol/L cutoff is a post-hoc exploratory finding, and its clinical utility cannot be generalized before external cohort validation. The associations of plasma Hcy and Cys with ICH prognosis were linear (both nonlinear *p* > 0.05).

### Predictive value of Hcy, Cys and Met levels for unfavorable prognosis in ICH patients

**Figure 8.**
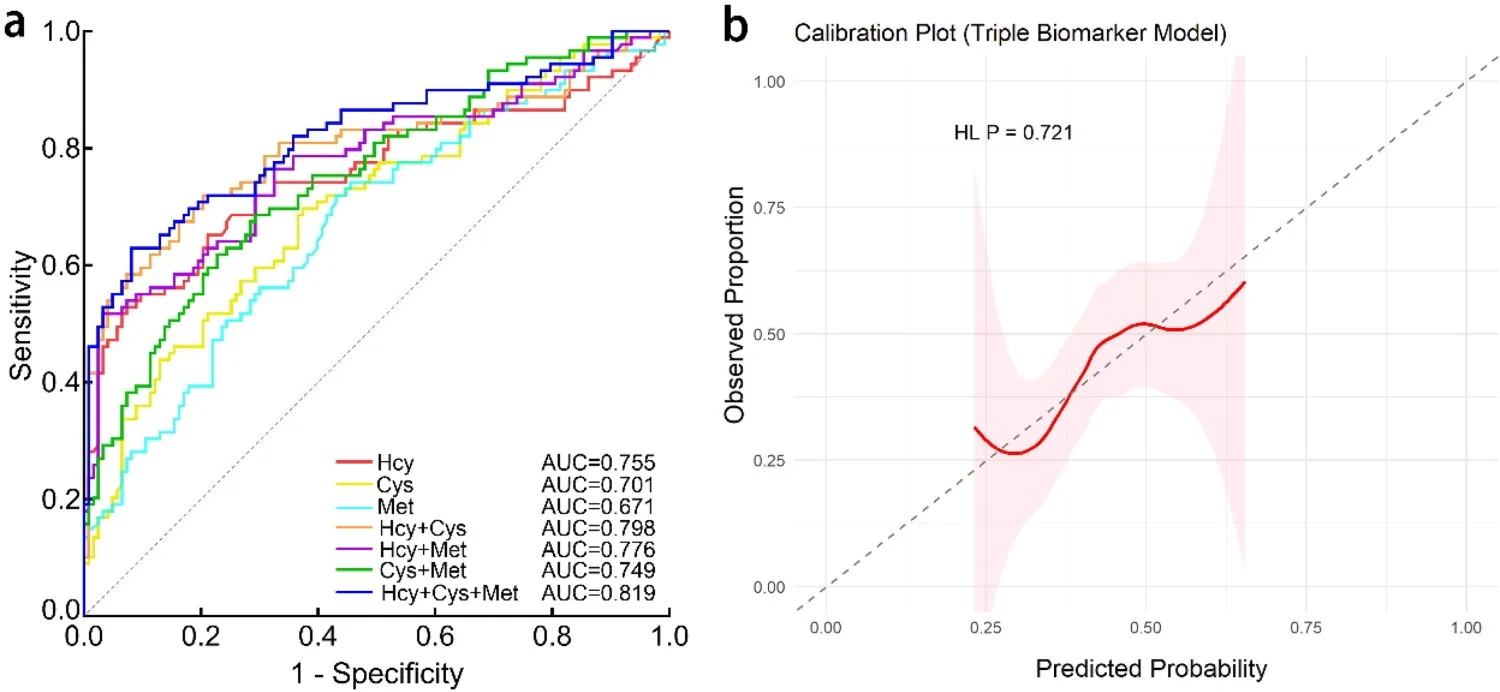
(a) ROC for homocysteine, cysteine and methionine in predicting ICH prognosis. (b) Calibration curve for model calibration evaluation (Hosmer-Lemeshow test).

Receiver operating characteristic (ROC) curves evaluating the predictive performance of Hcy, Cys, Met, and their combinations for unfavorable ICH prognosis are shown in Figure 8a. The AUC of Hcy for predicting unfavorable prognosis was 0.755 (95% CI: 0.679–0.822), with an optimal cutoff of 15.85 μmol/L, yielding a sensitivity of 52.81% and specificity of 92.68%. The AUC of Cys was 0.701 (95% CI: 0.629–0.773), with an optimal cutoff of 245.70 μmol/L (sensitivity 69.66%, specificity 62.60%). The AUC of Met was 0.671 (95% CI: 0.597–0.745), with an optimal cutoff of 21.21 μmol/L (sensitivity 71.91%, specificity 56.91%).

Pairwise combinations demonstrated improved predictive performance over individual biomarkers. The Hcy + Cys combination yielded an AUC of 0.798 (95% CI: 0.732–0.864), with sensitivity of 71.91% and specificity of 79.67%. The Hcy + Met combination achieved an AUC of 0.776 (95% CI: 0.709–0.843), with sensitivity of 51.69% and specificity of 96.75%. The Cys + Met combination resulted in an AUC of 0.749 (95% CI: 0.683–0.816), with sensitivity of 68.54% and specificity of 70.73%.

The triple-marker panel comprising Hcy, Cys, and Met demonstrated the strongest predictive performance, with an original AUC of 0.819 (95% CI: 0.758–0.880). After bootstrap internal validation (1,000 resamples), the optimism-corrected AUC was 0.807 (95% CI: 0.746–0.869). The calibration curve demonstrated good agreement between predicted probabilities and observed outcomes (Hosmer-Lemeshow test *p* = 0.721, Figure 8b).

**Figure 9.**
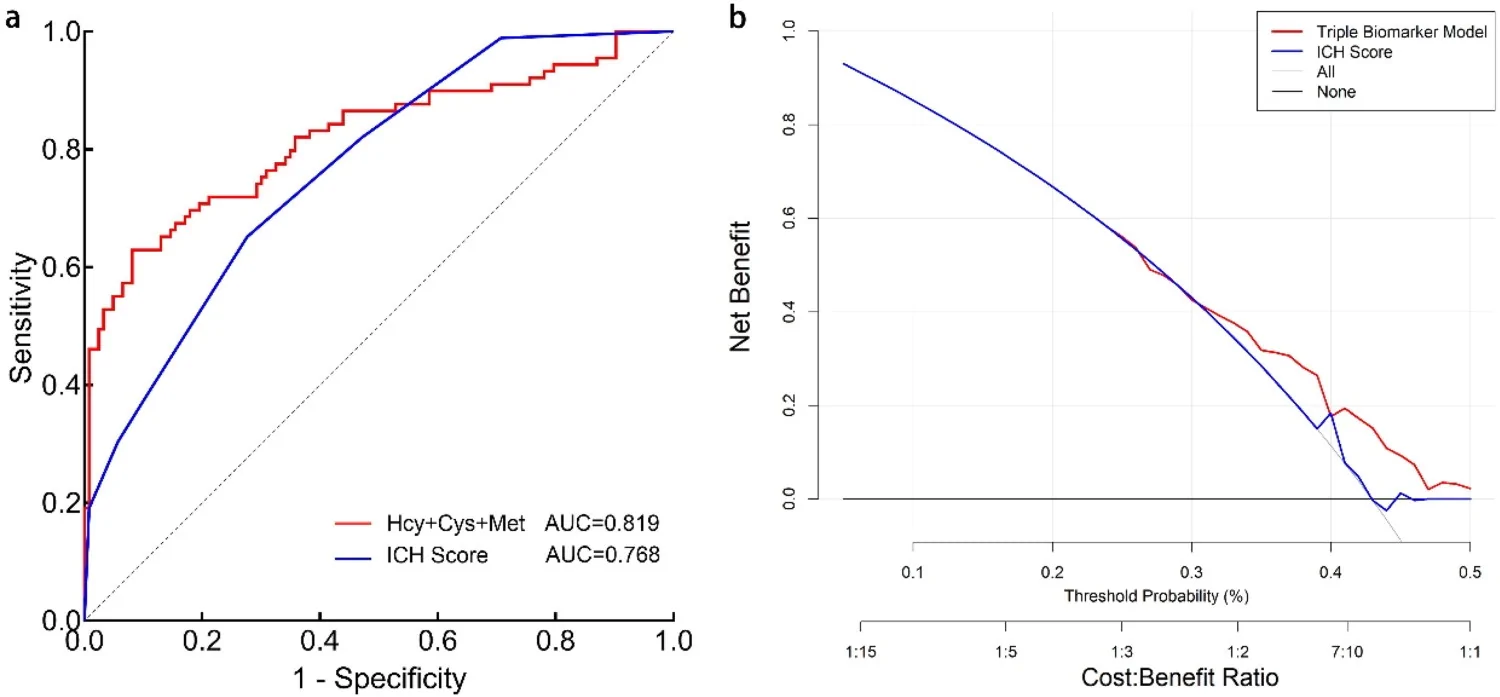
(a) Comparison between the classic ICH score with the triple-biomarker model (DeLong test). (b) Decision curve analysis (DCA) of the combined biomarker model and the classic ICH prognostic score. Hcy: homocysteine; Cys: cysteine; Met: methionine.

Compared with the classic ICH score (AUC = 0.768, 95% CI: 0.703–0.828), the triple-biomarker model exhibited significantly superior predictive efficiency (DeLong test *p* = 0.023), as shown in Figure 9a. Decision curve analysis (DCA) shown in Figure 9b further confirmed that the triple-biomarker model generated greater net clinical benefit than the ICH score and both extreme strategies (‘treat all’ and ‘treat none’) across a wide range of threshold probabilities (5%–40%).

**Figure 10.**
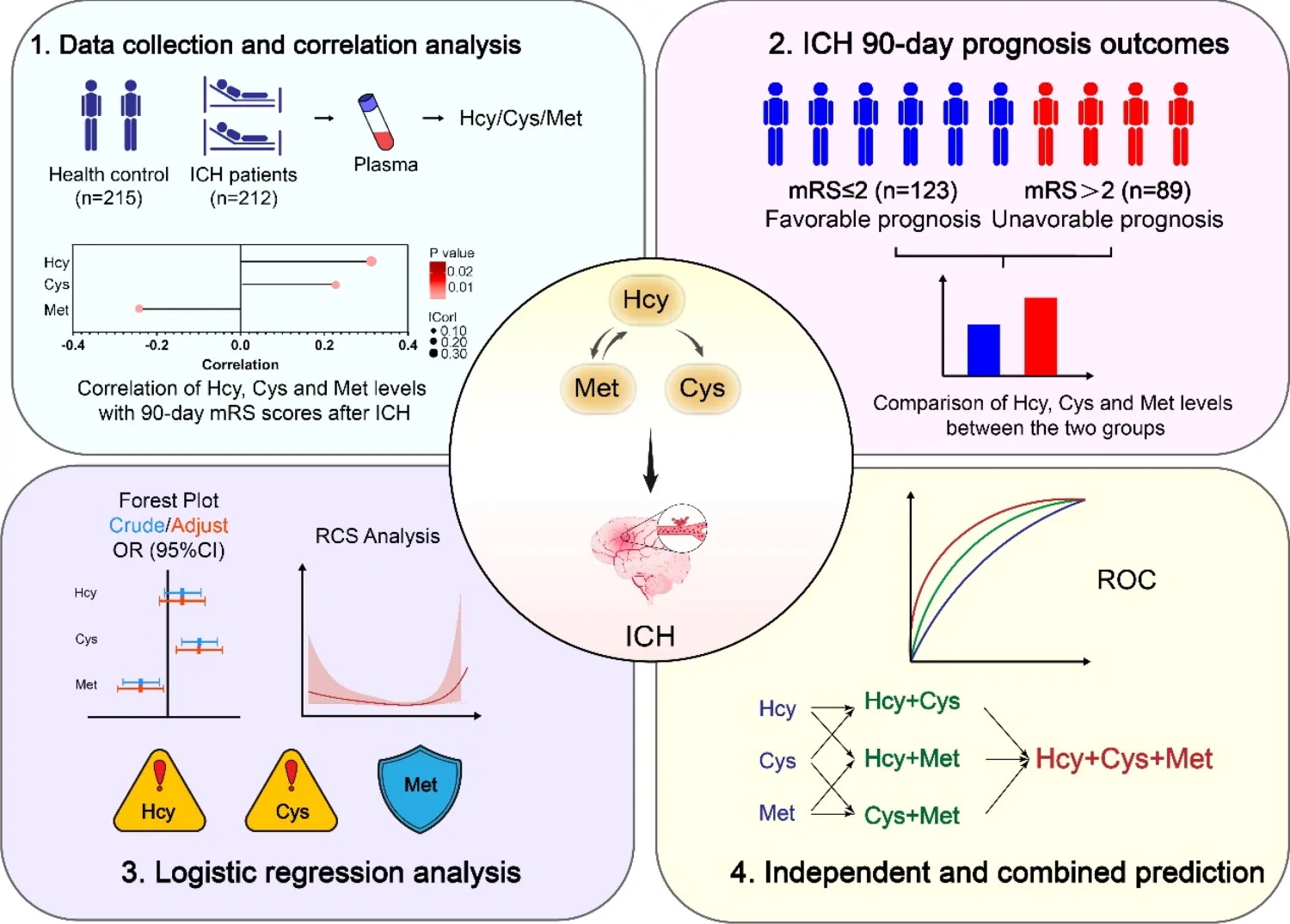
Graphical abstract of this study. Flow chart of the whole research design and main analysis contents: data collection → correlation analysis → logistic regression and RCS analysis → predictive model construction and verification. ICH: intracerebral hemorrhage; Hcy: homocysteine; Cys: cysteine; Met: methionine; RCS: restricted cubic spline analysis; ROC: receiver operating characteristic curve.

A graphical abstract summarizing the overall concept of this study is provided in Figure 10.

## Discussion

This study initially assessed plasma levels of Hcy, Cys and Met in patients with ICH and in healthy controls and analyzed the associations between these three SAAs and ICH risk. Furthermore, a 90-day follow-up was conducted to evaluate the relationships of Hcy, Cys and Met levels with clinical outcomes and to assess their individual and combined prognostic value.

Elevated Hcy levels are linked to a significantly increased risk of ICH compared to normal levels [23,24]. In our study, Hcy levels were notably elevated in the ICH patients than in healthy controls (11.77 vs. 8.43 μmol/L, *p* < 0.001). Elevated Hcy levels contribute to cerebrovascular dysfunction. The potential mechanisms are as follows: (1) Oxidative stress and antioxidant imbalance: Elevated Hcy levels stimulate excessive production of reactive oxygen species (ROS), reduce glutathione peroxidase (GPx) activity and trigger membrane lipid peroxidation. This process impairs endothelial function and damages the structural integrity of blood vessel walls [25,26]. (2) Vascular dysfunction: Hcy promotes the accumulation of asymmetric dimethylarginine (ADMA), which inhibits nitric oxide (NO) synthesis and leads to endothelial dysfunction [25]. Furthermore, Hcy induces the proliferation of vascular smooth muscle cells, thereby accelerating the development of atherosclerotic plaques [26]. (3) Inflammatory response: Hcy recruits macrophages into the vascular wall, promoting vascular inflammatory injury [27]. Hcy also stimulates the release of inflammatory mediators such as interleukin-6 (IL-6), interleukin-8 (IL-8) and high-sensitivity C-reactive protein (hs-CRP), which increase blood-brain barrier (BBB) permeability and disrupt cerebral vascular integrity [16].

Datas from the China National Stroke Center indicate that elevated Hcy levels may exacerbate severity of ICH, impair functional recovery, and predict an unfavorable prognosis in patients [15]. Hyperhomocysteinemia (HHcy) has been recognized as a distinct risk for unfavorable 90-day outcomes after ICH [16]. Our study demonstrated that individuals in the unfavorable prognosis group had markedly higher Hcy levels than those in the favorable prognosis group. Each 1-μmol/L increment in Hcy level was associated with a 23.5% higher risk of an unfavorable outcome. Patients in the highest Hcy quartile (>16.03 μmol/L) had a substantially increased risk of unfavorable prognosis (OR = 23.849), further confirming the correlation between elevated Hcy levels and unfavorable ICH prognosis. Elevated Hcy levels may impair neurite plasticity after ICH, slow neurite regeneration and delay motor recovery, thereby worsening prognosis [28]. Elevated Hcy levels may promote inflammation, which accelerates ICH progression and worsens prognosis [16]. Supplementation with folic acid, vitamin B6 and vitamin B12 can lower Hcy levels [29]. Although some studies suggest a protective effect of B vitamins supplementation against stroke [30,31], others report no significant benefits [32].

Cys, a metabolite of Hcy, plays a vital role in maintaining protein structure. Cysteine-altering variants are the core pathogenesis of CADASIL [33] and are highly associated with leukoaraiosis and lacunar infarcts [34], which elevate the risk of stroke. Cys reacts with glutamate and glycine to synthesize glutathione (GSH), a crucial molecule for the detoxification of reactive oxygen species (ROS) and the preservation of redox homeostasis [35,36]. Disruption of redox homeostasis can cause brain injury. Additionally, high levels of Cys are neurotoxic, which can lead to neuronal death, cerebral edema, and even epileptic seizures by activating ion channels, thereby exacerbating cerebrovascular injury [37]. Our analysis revealed that Cys levels were higher in the ICH patients than in healthy controls and significantly elevated in patients with unfavorable outcomes compared to those with favorable prognoses. Elevated Cys levels are an independent risk factor for an unfavorable prognosis in ICH. Each 1-μmol/L increment in Cys level was associated with 1.70% higher odds of an unfavorable prognosis. Moreover, patients in the fourth quartile of Cys (>278.53 μmol/L) have a significantly higher risk. Different from Hcy, current research on Cys in ICH is limited. Elevated Cys may disrupt the redox balance of brain tissue and produce direct neurotoxicity, which may contribute to poor functional recovery of ICH patients. This study supplements the clinical evidence of Cys in the field of ICH prognosis.

Met is an essential amino acid and precursor for Hcy synthesis. We observed a nonlinear correlational trend between Met levels and ICH prognosis. At adequate physiological levels, which is within the range below 32.88 μmol/L in our study, Met may confer protective effects by maintaining antioxidant effect [20] or mitochondrial function [38]. It also has a potential effect on cerebrovascular repair [39]. Prior literature suggests extreme Met overload might promote cerebrovascular injury *via* HHcy [40,41]. Met can be oxidized by ROS to form methionine sulfoxide. This compound exhibits a notable correlation with an elevated hemorrhagic risk in moyamoya disease [42,43]. Such pathogenic damage was not directly tested in our observational analysis, so causal inference cannot be drawn from the present data. Met levels in all samples were within normal reference ranges in our study. Met levels were elevated in patients with ICH compared to healthy controls and were significantly higher in patients with favorable prognosis than in those with unfavorable prognosis. Each 1-μmol/L increase in Met level was associated with 7.6% reduced risk of unfavorable prognosis. The 32.88 μmol/L inflection point of Met concentration was obtained through post-hoc exploratory analysis of this single-center retrospective dataset, and further multicenter prospective work is warranted to establish its potential utility as a surveillance index for plasma Met among ICH sufferers.

Hcy showed the strongest individual predictive value. Combined models performed better than single indicator, and combining Hcy, Cys and Met achieved the highest accuracy. (AUC = 0.819). These findings highlight that the triple combination of Hcy, Cys and Met has greater clinical value, providing a more comprehensive risk assessment of unfavorable prognosis in ICH patients. Compared with the traditional ICH clinical scoring system, this triple-biomarker model has higher predictive accuracy, and can realize rapid objective detection within 24 h after admission, which makes up for the deficiency of subjective scoring. DCA indicated promising net benefit within the studied probability spectrum, yet further external validation is essential to substantiate its durable clinical performance across diverse ICH populations.

Our study bears several limitations: (1) As a single-center retrospective investigation, our findings may be subject to regional selection bias, requiring prospective multicenter trials to confirm external validity. (2) Plasma biomarkers were measured only 24 h after admission; residual confounding from acute hemorrhagic stress cannot be fully ruled out, and serial multi-timepoint sampling is warranted in further research. (3) Surgically treated ICH patients were excluded, so our results cannot be generalized to severe surgical cases. (4) Being an observational analysis, this work merely identifies correlations instead of causal relationships between the three sulfur amino acids and ICH prognosis. (5) Critical confounders (admission NIHSS, baseline renal function, statin use) were not documented in the raw dataset, resulting in unadjusted residual bias in multivariate regression. (6) Sample size evaluation was conducted post-hoc rather than prospectively pre-specified, compromising methodological rigor. (7) The 32.88 μmol/L Met inflection point derived from RCS is a post-hoc exploratory outcome without pre-defined protocol, whose clinical translational value awaits validation in independent cohorts. (8) Only internal bootstrap validation was implemented, with no external cohorts to test model generalizability.

## Conclusion

Our study demonstrated that plasma levels of Hcy, Cys and Met are significantly associated with 90-day prognosis in ICH patients. Elevated levels of Hcy and Cys were independent risk factors for an unfavorable prognosis in ICH patients, while adequate Met concentrations below an exploratory post-hoc inflection point (32.88 μmol/L) were linked to lower odds of poor recovery. As an observational study, we only identified epidemiological associations rather than causal relationships. Interventional trials are required to test whether modulating sulfur amino acid levels improves ICH functional recovery. The triple-biomarker panel achieved superior predictive performance compared with single or dual biomarker models based on internal bootstrap validation, and external multi-center validation is mandatory to confirm its generalizability for clinical risk stratification.

## Data Availability

All data employed in this study can be obtained from the corresponding author upon formal and justified request.
